# Impact of a multifaceted intervention including supportive care order sentence implementation on outpatient antibiotic prescribing for upper respiratory tract infections

**DOI:** 10.1017/ash.2025.10135

**Published:** 2025-09-10

**Authors:** Ashlyn M. Kiebach, Lauren R. Stonerock, Tara E. McAlpine, Julie A. Earby, Jessica A. Benzer, Nnaemeka E. Egwuatu, Andrew P. Jameson, Lisa E. Dumkow

**Affiliations:** 1 Department of Pharmacy, Trinity Health Grand Rapids Hospital, Grand Rapids, MI, USA; 2 Trinity Health Alliance, Muskegon, MI, USA; 3 Department of Infectious Diseases, Trinity Health Grand Rapids Hospital, Grand Rapids, MI, USA; 4 Michigan State University College of Human Medicine, Grand Rapids, MI, USA

## Abstract

**Objective:**

Compare the incidence of antibiotic prescribing for bronchitis and sinusitis before and after implementation of a multifaceted outpatient stewardship intervention.

**Design:**

Retrospective, quasi-experimental study.

**Setting:**

Three primary care clinics within a Michigan health system.

**Patients:**

Age 3 months and older who were diagnosed with acute bronchitis or rhinosinusitis.

**Intervention:**

Provider education paired with electronic health record order sentences for supportive care were implemented in September 2024. Patients diagnosed with bronchitis or sinusitis between October 2023 and January 2024 were included in the pre-intervention group (pre-ASP) while patients diagnosed between October 2024 and January 2025 were in the post-implementation group (post-ASP).

**Results:**

Total antibiotic prescribing for acute bronchitis and rhinosinusitis decreased significantly following the intervention (pre-ASP 65.6% vs post-ASP 53.9%, *P* = .024) and was driven by a significant reduction in prescribing for bronchitis post-ASP (36.7% vs 21.1%, *P* = .021). Antibiotic prescribing for rhinosinusitis decreased but did not reach statistical significance (94.4% vs 86.7%, *P* = .074). The relative reduction in antibiotic prescribing in the presence of a supportive care prescription for acute bronchitis was 51.2% (37.1% vs 18.1%, *P* = .018).

**Conclusions:**

Supportive care order sentence implementation paired with provider education may be an effective outpatient stewardship practice to reduce antibiotic prescribing for URI.

## Introduction

Upper respiratory tract infections (URIs), such as acute bronchitis and rhinosinusitis, contribute to over 100 million ambulatory care visits in the United States annually.^1^ While it is estimated that more than 90% of acute bronchitis and rhinosinusitis cases are caused by viral pathogens, antibiotics are prescribed in nearly 70% and 80% of outpatient visits.^2–4^ Recognizing the negative impacts of poor outpatient antimicrobial stewardship, health plans in the United States have begun focusing on strategies to incentivize the avoidance of unnecessary antibiotic prescribing in their Healthcare Effectiveness Data and Information Set (HEDIS) measures.^5–7^ These pay-for-performance measures are utilized by more than 90% of U.S. health plans to promote accountability and improve the quality of care for Tier 3 (antibiotics almost never indicated) and Tier 2 (antibiotic sometimes indicated) infections.^8,9^ Despite HEDIS measures offering financial incentives to minimize antibiotic prescribing for viral URIs, many sites across the country continue to underperform, illustrating the need for outpatient stewardship interventions which incorporate both education and clinical tools to help reduce unnecessary antibiotic prescribing.

Patients presenting with URI symptoms in outpatient settings often expect to be provided with a prescription at the conclusion of their visit. Previous studies have demonstrated that the prescribing of nonantibiotic, supportive care therapy, such as decongestants or antitussives, can result in comparable patient satisfaction scores to the prescribing of antibiotics for both adults and pediatric patients.^10,11^ Addressing patient expectations and providing education related to supportive care therapies requires providers to maintain their knowledge of these medications which can take up a considerable amount of visit time. Antimicrobial stewardship interventions that include provider education and electronic health record (EHR) tools can streamline computerized order entry while providing patients the counseling they need to increase the use of supportive care therapies.^12^ The purpose of this study was to assess the change in antibiotic prescribing for acute bronchitis and acute rhinosinusitis following the implementation of a multifaceted outpatient intervention including interprofessional education coupled with supportive care order sentence development and patient education tools within the EHR.

## Methods

### Study site and Antimicrobial Stewardship Program

This study was conducted at three practices within a large primary care network in West Michigan. This network consists of 17 locations across the Grand Rapids region and is affiliated with a 300-bed community teaching hospital. The provider staff at the three selected family medicine offices included 14 physicians and 10 advanced practice providers (APPs). Embedded ambulatory care pharmacists support these primary care practices; however, their daily activities center around chronic disease state management, and they do not have dedicated time toward antimicrobial stewardship.

The health system has an Antimicrobial Stewardship Program (ASP) that was established in July 2013 and is led by 1.0 full-time equivalent (FTE) infectious diseases pharmacist and .2 FTE infectious diseases physician. The ASP annually publishes an outpatient antibiogram with empiric therapy guidelines to provide treatment recommendations for ambulatory antimicrobial therapy based on existing guidelines.^13–15^ Outpatient ASP initiatives focused on improving primary care prescribing began in August 2017 and initially centered around audit-and-feedback provided by the ambulatory care pharmacists. Given the increasing demand for ambulatory care pharmacists in providing disease state management and the constraints on their availability, the ASP shifted focus to prioritizing the optimization of the EHR through antimicrobial order sentence development and targeted education. This approach enabled broader and more sustainable antimicrobial stewardship across the health system, offering a lower-effort alternative to relying solely on limited pharmacist resources. While this strategy improved guideline-concordant antibiotic prescribing (agent, dose, duration), this strategy is unable to target decreasing antimicrobial prescriptions for indications where antibiotics should not be prescribed.^16^

This was a retrospective, quasi-experimental study designed to evaluate whether the implementation of supportive care order sentences for the treatment of acute bronchitis and rhinosinusitis within the EHR paired with education would reduce the incidence of antibiotic prescribing for these conditions within three selected primary care clinics. Two time periods were designated for comparison. Patients diagnosed with acute bronchitis or rhinosinusitis from October 1, 2023, through January 31, 2024, were included in the pre-order-sentence-implementation group (pre-ASP), while the post-order-sentence implementation group (post-ASP) included patients diagnosed for the same indications from October 1, 2024, through January 31, 2025. The three clinics were selected to pilot the intervention based on their 2023 HEDIS performance (one high, one medium, and one low performing clinic selected). Patients were eligible for study inclusion if they were aged 3 months or older and were diagnosed with acute bronchitis or acute rhinosinusitis during an in-person or telemedicine encounter. Patients were excluded who had encounters that resulted in hospitalization, were receiving hospice care, were receiving antibiotics prior to the visit or for a concomitant infection, had an unspecified duration of cough or cough greater than three weeks duration, had chronic sinusitis, or received antibiotics from a provider not included in the intervention. A report was generated from the EHR of all patients diagnosed with the selected indications during the eligible time periods. The list was then randomized, and patients were screened for eligibility by the primary investigator until the desired sample size was met. This study was approved by the local Institutional Review Board.

### Education and EHR order sentence optimization intervention

Order sentences for supportive care were built by the ambulatory care pharmacist supervisor within the Epic outpatient EHR providing recommendations for common therapies utilized to manage URI symptoms in both pediatrics and adults. The order sentences were organized by age group (pediatric or adult) and by indication (bronchitis and rhinosinusitis); available supportive care agents were listed in alphabetical order by class and not clinical preference. Selecting an agent would populate a corresponding prescription order to electronically prescribe with an appropriate dose, frequency, and duration of therapy (Supplementary Figure 1). Order sentence implementation occurred in September 2024; each clinic team had an hour-long presentation conducted by the primary investigator along with the ASP pharmacist, embedded clinical ambulatory pharmacist, and ambulatory pharmacist supervisor. The presentation centered around the significance of outpatient antimicrobial stewardship, supportive care as an alternative to antibiotics for viral conditions, and guidance on utilization of order sentences. Detailed written instructions were shared with each clinic to guide users on how to find and to utilize the supportive care order sentences. In this education, providers were also redirected to the preexisting antimicrobial order sentences developed for the treatment of acute bacterial rhinosinusitis based on the institution’s empiric therapy guidelines. Prewritten blocks of text known as a “SmartPhrase” in Epic EHR systems were shared with the providers to assist with education to patients about available non-pharmacologic supportive care treatments for each indication based on age group (Supplementary Figure 2). Lastly, each clinic was offered a patient-facing flyer to share education on the importance of appropriate antibiotic use and the harms of poor antimicrobial stewardship (Supplementary Figure 3).

### Study endpoints

The primary outcome of this study was to compare the proportion of patients receiving antibiotics prescribed for acute bronchitis and acute rhinosinusitis before and after implementation of supportive care order sentences and education. Secondary outcomes included evaluating supportive care agents recommended or prescribed and patient outcomes, which included adverse drug events and follow-up encounters for the same indication due to symptoms not improving within 7 and 30 days. We additionally evaluated the incidence of guideline-concordant antibiotic prescribing for acute rhinosinusitis defined as appropriate drug, dose, frequency, and duration based on the institution’s outpatient empiric antimicrobial therapy guidelines.

### Statistical analysis

Assuming a baseline rate of antibiotic prescribing for these indications to be 30% based on previous antibiotic prescribing rates for acute bronchitis from the largest pilot clinic in this study, a sample size calculation of 120 subjects per clinic would be needed to detect a 5% difference in the primary outcome using a two-sided alpha of .05 and a power of 80%. The primary outcome of total antibiotic prescribing for these indications was evaluated with the Chi Squared test. Other secondary endpoints consisting of categorical data were analyzed with Chi Squared or Fischer’s exact test. The student’s t-test or Mann–Whitney U test were used, as appropriate, to compare interval data. All statistical analyses were performed using SPSS software, version 22 (IBM, Corporation, Armonk, New York).

## Results

**Figure 1.**
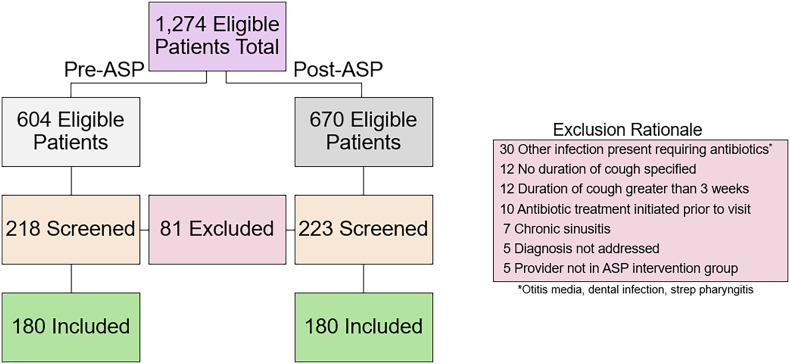
Patient eligibility and screening process. ASP, Antimicrobial Stewardship Program.

A total of 1,274 patients aged 3 months and older diagnosed with acute bronchitis or rhinosinusitis were identified from the study periods. Patients were screened until 360 patients met eligibility criteria (Figure 1). The most common reason for exclusion was the utilization of antibiotics for concomitant diagnoses. Patient characteristics are described in Table 1. Most patients across both pre-ASP and post-ASP groups were non-Hispanic white females with an average age of 45 years, median Charlson Comorbidity Index of 1, and did not have an underlying respiratory condition. There was no difference in age, co-morbidities, race, or ethnicity between the pre-ASP and post-ASP groups. Most encounters were conducted in-person in both groups (pre-ASP 92.8% vs post-ASP 90.6%, *P* = .385). The median durations of symptoms for each indication were not different across groups for each indication: 7 days for bronchitis (pre-ASP 7 [IQR 4–14] and post-ASP 7 [4–10.25]) and approximately 11 days for rhinosinusitis (pre-ASP 11.50 [IQR 7.00–16.75] and post-ASP 11 [IQR 7–16.50]).

**Table 1.** Patient characteristics

Patient characteristics	Total (*n* = 360)
Female sex, *n* (%)	270 (75.0)
Age, average ± SD	45.5 ± 22.0
Less than 18 years	46 (12.8)
18 to 64 years	231 (64.2)
65 years or greater	83 (23.1)
**Race**, *n* (%)	
White	283 (78.6)
Black or African American	37 (10.3)
Unknown (selected by patient)	11 (3.1)
Asian	10 (2.8)
Unable to determine	9 (2.5)
Other	5 (1.4)
American Indian or Alaska Native	4 (1.1)
Native Hawaiian or Other Pacific Islander	1 (.3)
**Ethnicity,** *n* (%)	
Hispanic or Latino	34 (9.4)
**Insured**, *n* (%)	
Access to Prescription Insurance	356 (98.9)
Commercial / Private	219 (60.8)
Medicare	94 (26.1)
Medicaid	43 (11.9)
**Co-morbidities**	
Charlson Comorbidity Index, Median (IQR)	1 (0, 2)
Asthma, *n* (%)	43 (11.9)
COPD, *n* (%)	9 (2.5)
Other respiratory disease, *n* (%)	6 (1.7)
**Allergies**, *n* (%)	
Beta-Lactam allergies	31 (8.6)
Intolerance reaction	2 (0.6)
Non-severe reaction	18 (5.0)
Severe reaction	3 (0.8)
Unknown reaction	8 (2.2)

### Antibiotic prescribing outcomes

Total antibiotic prescribing for acute bronchitis and rhinosinusitis decreased significantly following the implementation of supportive care order sentences and education (pre-ASP 65.6% vs post-ASP 53.9%, *P* = .024). There was a significant reduction in antibiotic prescribing for bronchitis (36.7% vs 21.1%, *P* = .021) with each clinic demonstrating a similar reduction in prescribing, regardless of baseline HEDIS performance (Table 2). Of the patients who were prescribed antibiotics for bronchitis, the median duration decreased by 2 days in the post-ASP period (7 [IQR 5–7] vs 5 [IQR 5–7], *P* = .174). For acute rhinosinusitis, antibiotic use was indicated in 55.6% of pre-ASP and 56.7% of post-ASP cases (*P* = .881). Antibiotic prescribing was high across both groups with a nonsignificant reduction in prescribing in the post-ASP group following the intervention (pre-ASP 94.4% vs post-ASP 86.7%, *P* = .074) which was consistent across pilot clinics. There was no difference in guideline-concordant antibiotic selection (pre-ASP 67.1% vs post-ASP 75.3%, *P* = .247), dose (pre-ASP 96.5% vs post-ASP 97.4%, *P* = .732), and duration (pre-ASP 62.4% vs post-ASP 65%, *P* = .733) for rhinosinusitis following the intervention. The median duration of antibiotics prescribed in both groups was seven days (pre-ASP 7 [IQR 7–10] vs post-ASP 7 [IQR 5–10] days, *P* = .274). There was no difference in the percent of patients following up for ongoing URI symptoms without improvement at 7 (pre-ASP 7.2% vs post-ASP 7.8%, *P* = .841) and 30 days (pre-ASP 12.2% vs post-ASP 9.4%, *P* = .397) between groups.

**Table 2.** Antibiotic prescribing across three pilot clinics

Indication	Location	Antibiotics ordered (%)	
		Pre-ASP (*n* = 30)	Post-ASP (*n* = 30)
Acute bronchitis, *n* (%)	Clinic A	21 (70.0)	17 (56.7)
	Clinic B	7 (23.3)	2 (6.7)
	Clinic C	5 (16.7)	0 (0)
Acute rhinosinusitis, *n* (%)	Clinic A	28 (93.3)	26 (86.7)
	Clinic B	29 (96.7)	26 (86.7)
	Clinic C	28 (93.3)	26 (86.7)

Baseline HEDIS performance: Clinic A, Low; Clinic B, Medium; Clinic C, High.

### Supportive care prescribing outcomes

**Figure 2.**
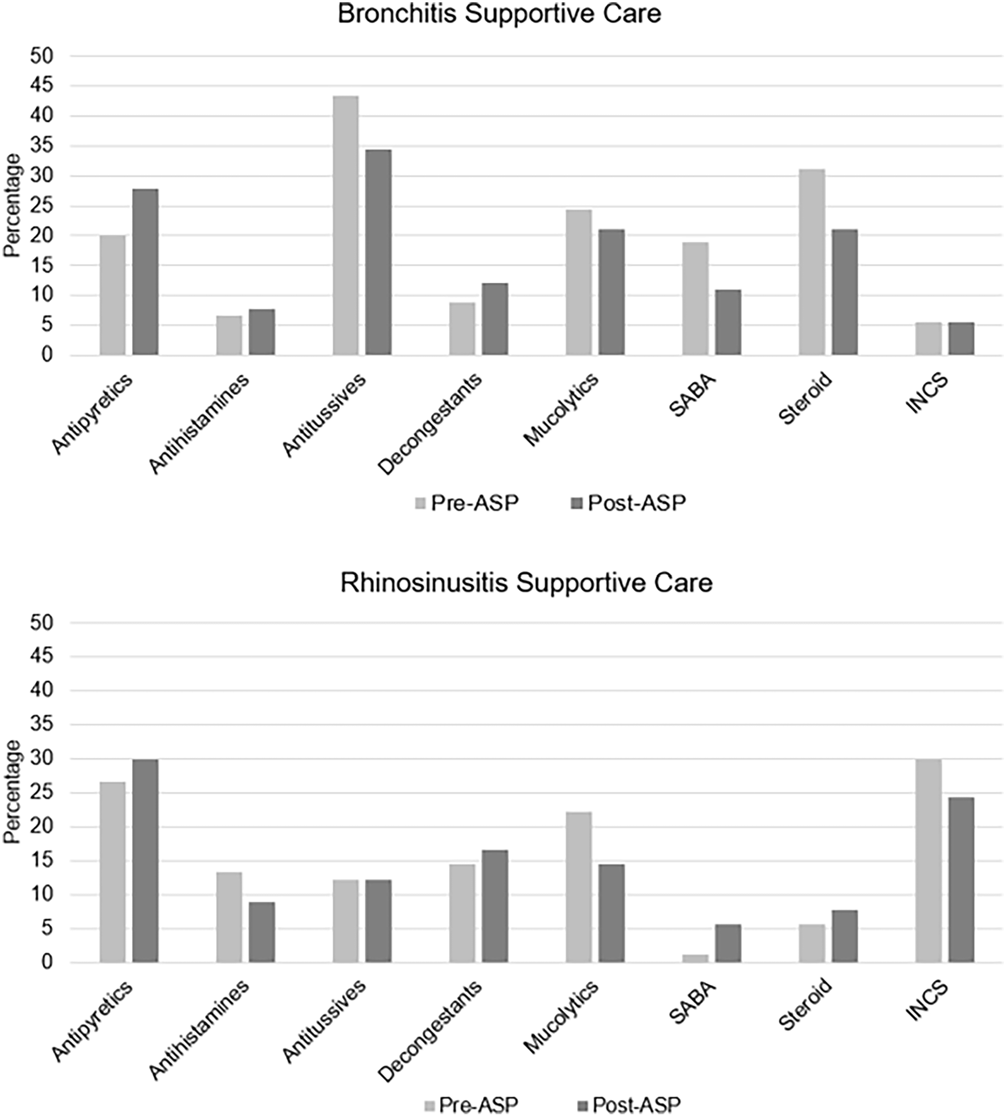
Supportive care recommendation trends. Incidence of supportive care therapeutic class recommended (as determined by encounter documentation alone and/or outpatient prescription ordered during encounter). SABA, short-acting beta-2 agonist; INCS, intranasal corticosteroid.

Supportive care prescribing trends for each indication are displayed in Figure 2. There was no difference in the percentage of patients receiving supportive care medications between groups (pre-ASP 69.4% vs post-ASP 67.2%, *P* = .650). There was a statistically significant reduction in antibiotic prescribing across both indications when supportive care was recommended following ASP implementation (pre-ASP 61.6% vs post-ASP 47.9%, *P* = .031). The relative reduction in antibiotic prescribing in the presence of supportive care for acute bronchitis was 51.2% (pre-ASP 37.1% vs post-ASP 18.1%, *P* = .018). The reduction in antibiotic prescribing for acute rhinosinusitis in the presence of supportive care was not significant (pre-ASP 92.7% vs post-ASP 80.7%, *P* = .062).

## Discussion

Our findings indicate that a multifaceted outpatient antimicrobial stewardship intervention including interprofessional education, patient-facing educational materials, and the implementation of EHR order sentences for supportive care significantly reduces antibiotic prescribing for URIs. Additionally, the reduction in antibiotic prescribing did not result in an increase in the need for follow-up for ongoing or worsening URI symptoms at 7 or 30 days demonstrating that the intervention was not only effective but also did not result in worse patient outcomes. The clinical significance of this work in supporting outpatient antimicrobial stewardship lies in the overall simplicity of the intervention. The use of the EHR to conduct the bulk of the intervention required minimal time commitment with the potential for high impact.

The significant reduction in antibiotic prescribing for URI we observed was most notable for bronchitis with only a moderate reduction observed for rhinosinusitis. This may be largely attributable to clear guidance that bronchitis almost never warrants antimicrobial therapy as a Tier 3 infection. While previous studies have focused on EHR optimization with antibiotic order sentences as a means of improving outpatient antimicrobial prescribing (agent, dose, duration), we believe this is the first study to evaluate EHR order sentences for supportive care therapies as a method to reduce overall antibiotic prescribing. Antibiotic order sentences are important in supporting guideline-concordant prescribing for Tier 1 and Tier 2 indications; however, they likely have no impact on Tier 3 indications where antimicrobials are not warranted. Previous studies have demonstrated that audit-and-feedback is effective in reducing unnecessary antibiotic prescribing for Tier 3 diagnoses; however, these interventions are time-intensive and may prove unsustainable over time.^17–23^ Harrigan et al. evaluated the impact of a multifaceted outpatient intervention targeting viral URIs and saw a significant reduction in antibiotic prescribing; however, at the conclusion of the funding period the intervention was stopped and prescribing rates rebounded significantly.^24^ Our intervention involving education and development of EHR tools to promote supportive care over antibiotic therapy for acute bronchitis may be a more easily sustainable outpatient stewardship strategy compared to audit-and-feedback. Notably, while our low performing site saw a similar absolute reduction in antibiotic prescribing for bronchitis, their overall prescribing rate remained over 50% and well above the rate of our moderate and high performing sites. In outpatient settings where resources are limited, stewardship programs may benefit from prioritizing automated interventions such as supportive care order sentence development. However, program leaders should be mindful that resource allocation to support the addition of other, more time-intensive strategies, such as audit-and-feedback or scorecard incentives, may be necessary to bring low performing sites to the level of higher performing clinics.

Prescribing for rhinosinusitis decreased but remained higher than 80% across all clinics following the intervention, underscoring the difficulty in developing successful antimicrobial stewardship interventions for Tier 2 indications. Approximately 50% of patients in our study met the CDC criteria for bacterial rhinosinusitis which is similar to previous investigations in primary care; despite this, over-treatment still exceeded 30% in both the pre- and post-ASP groups.^25,26^ Previous antimicrobial stewardship interventions targeting rhinosinusitis have found success with more resource-intensive strategies such as audit-and-feedback; however, the impact of low effort, EHR automated interventions have shown mixed results. An observational cohort study by Ginzburg et al. evaluated the impact of an integrated best practice alert (BPA) in the EHR of a single health clinic.^27^ The BPA provided antibiotic regimen recommendations and also reminded prescribers diagnosing sinusitis that viral causes were most likely and antibiotics may not be warranted. The BPA intervention demonstrated a significant reduction in antibiotic prescribing for acute rhinosinusitis from 86.3% to 61.7% (relative risk: .69, 95% CI .51–.95). Hansen et al. implemented a similar BPA for sinusitis across 117 Midwest primary care clinics.^28^ In contrast to the findings of Ginzburg, the authors found no difference in antibiotic prescribing despite the BPA firing in 7,780 visits (pre-BPA 94.8% vs post-BPA 94.3%, *P* = .152). While low-effort interventions are attractive for Tier 2 indications, caution and careful monitoring of their impacts are needed to identify if more resource-intensive strategies are warranted.

There are limitations to our study which must be considered. First, the data were collected retrospectively which relies on the accuracy of prescriber diagnosis and documentation. Prescribers differed slightly between groups; while we intended to include the same providers a few left the practice, and their data were only included in the Pre-ASP group. Additionally, while our EHR is connected to several other health systems, it is possible that patients sought follow-up within a healthcare setting that was unable to be captured by our EHR. While our study did not find a statistically significant reduction in antibiotic prescribing postintervention for acute rhinosinusitis, our sample size was limited, and we were not powered for sub-group analyses and may have shown significance with a larger study population. Additionally, we applied this intervention across a wide age range and recognize that there may be distinct differences in how providers diagnose and treat pediatric versus adult URIs; however, we felt this was warranted as HEDIS measures are applied similarly to both pediatric and adult patients. While we created a patient-facing flier to help provide education and address patient expectations related to antibiotics for URI, we were unable to measure how often these were utilized by providers or measure their impact on patient perception and requests for antibiotics. We were unexpectedly unable to access data on supportive care order sentence and SmartPhrase utilization from EHR due to functionality limitations associated with single-user owned order sentences. Therefore, we were unable to determine if our findings are driven by education alone or the combination of education with the EHR supportive care prescribing tools. While the absence of a significant difference in total supportive care prescribing following the intervention may suggest that education played a large role, the observed 51% relative reduction in antibiotic prescribing in the presence of supportive care for bronchitis is likely higher than would be expected from an educational session alone. It is also possible that prescribers recommended supportive care without a prescription or documentation of the recommendation within the encounter. Lastly, we acknowledge that not all EHR systems can implement customized order sentences which could limit the application of these findings broadly into clinical practice. Despite these limitations, our findings provide insight into a novel, low-effort, outpatient antimicrobial stewardship approach focused on optimization of the EHR to support prescriber recommendations for supportive care in the place of antibiotic therapy which can have a significant impact on reducing unnecessary antibiotic prescribing for URI.

Provider education paired with the development of EHR order sentences for supportive care therapies offers a relatively low effort but high yield outpatient antibiotic stewardship intervention resulting in a significant reduction in unnecessary antibiotic prescribing for URIs, particularly acute bronchitis. Future research is needed to define the optimal strategies and resources necessary to reduce antibiotic prescribing for Tier 2 respiratory diagnoses and in low performing clinics.

## Supporting information

10.1017/ash.2025.10135.sm001 Kiebach et al. supplementary material Kiebach et al. supplementary material

## Data Availability

The data that supports the findings of this study are available from the corresponding author upon reasonable request.

## Supplementary material

The supplementary material for this article can be found at https://doi.org/10.1017/ash.2025.10135.
