# Supplementary material for: Impact of a multifaceted intervention including supportive care order sentence implementation on outpatient antibiotic prescribing for upper respiratory tract infections
Source: Antimicrob Steward Healthc Epidemiol. 2025 Sep 10;5(1):e206. doi: 10.1017/ash.2025.10135 (PMC12451796; doi:10.1017/ash.2025.10135)
Supplement: Kiebach et al. supplementary material [file S2732494X25101356sup001.pdf]

Supplementary Materials

Supplementary Figure 1 – Supportive Care Order Sentences and Populated Order Example

Acute Bronchitis Supportive Care

Antipyretics & Pain Management

- ☐ acetaminophen (TYLENOL) tablet 500 mg (1000mg Q 8hr)
- ☐ acetaminophen (TYLENOL) tablet 500 mg (500mg Q 4hr)
- ☐ ibuprofen (MOTRIN) tablet 400 mg (400mg Q 4hr)
- ☐ ibuprofen (MOTRIN) tablet 600 mg (600mg Q 6hr)

Cough Suppressants

- ☐ benzonatate (TESSALON) capsule 100 mg
- ☐ benzonatate (TESSALON) capsule 200 mg
- ☐ dextromethorphan polistirex ER (DELSYM) suspension 6 mg/mL

Bronchospasm Management

- ☐ albuterol (PROAIR HFA ; PROVENTIL HFA ; VENTOLIN HFA) inhaler 108 (90 BASE) MCG/ACT
- ☐ albuterol nebulizer solution 2.5 mg/3 mL (0.083%)

Acute Rhinosinusitis Supportive Care

Antipyretics & Pain Management

- ☐ acetaminophen (TYLENOL) tablet 500 mg
- ☐ ibuprofen (MOTRIN) tablet 400 mg

Decongestants

- ☐ oxymetazoline (AFRIN) nasal spray 0.05%
- ☐ phenylephrine (SUDAFED PE) tablet 10 mg
- ☐ pseudoephedrine (SUDAFED) 12 hr tablet 120 mg
- ☐ pseudoephedrine (SUDAFED) tablet 60 mg

Mucolytics

- ☐ guaiFENesin (MUCINEX) 12 hr tablet 600 mg
- ☐ guaiFENesin tablet 200 mg

Antihistamines

- ☐ cetirizine (ZyrTEC) tablet 10 mg
- ☐ loratadine (CLARITIN) tablet 10 mg

Cough Suppressants

- ☐ benzonatate (TESSALON) capsule 100 mg
- ☐ benzonatate (TESSALON) capsule 200 mg
- ☐ dextromethorphan polistirex ER (DELSYM) suspension 6 mg/mL

Acute Bronchitis Supportive Care

Antipyretics & Pain Management

- ☐ acetaminophen (TYLENOL) tablet 500 mg
- ☐ acetaminophen (TYLENOL) tablet 500 mg
- ☐ ibuprofen (MOTRIN) tablet 400 mg
- ☐ ibuprofen (MOTRIN) tablet 600 mg

Cough Suppressants

- ☐ benzonatate (TESSALON) capsule 100 mg
- ☐ benzonatate (TESSALON) capsule 200 mg
- ☐ dextromethorphan polistirex ER (DELSYM) suspension 6 mg/mL

Bronchospasm Management

- ☐ albuterol (PROAIR HFA ; PROVENTIL HFA ; VENTOLIN HFA) inhaler 108 (90 BASE) MCG/ACT
- ☐ albuterol nebulizer solution 2.5 mg/3 mL (0.083%)

Acute Rhinosinusitis Supportive Care

Antipyretics & Pain Management

- ☐ acetaminophen (TYLENOL) tablet 500 mg
- ☐ ibuprofen (MOTRIN) tablet 400 mg

Decongestants

- ☐ oxymetazoline (AFRIN) nasal spray 0.05%
- ☐ phenylephrine (SUDAFED PE) tablet 10 mg
- ☐ pseudoephedrine (SUDAFED) 12 hr tablet 120 mg
- ☐ pseudoephedrine (SUDAFED) tablet 60 mg

Mucolytics

- ☐ guaiFENesin (MUCINEX) 12 hr tablet 600 mg
- ☐ guaiFENesin tablet 200 mg

Antihistamines

- ☐ cetirizine (ZyrTEC) tablet 10 mg
- ☐ loratadine (CLARITIN) tablet 10 mg

Cough Suppressants

- ☐ benzonatate (TESSALON) capsule 100 mg
- ☐ benzonatate (TESSALON) capsule 200 mg
- ☐ dextromethorphan polistirex ER (DELSYM) suspension 6 mg/mL

Pediatric Acute Bronchitis/Bronchiolitis Supportive Care

Antipyretics & Pain Management

- ☐ acetaminophen (TYLENOL) chewable tablet 160 mg
- ☐ acetaminophen (TYLENOL) liquid 160 mg/5mL
- ☐ acetaminophen (TYLENOL) tablet 325 mg
- ☐ ibuprofen (ADVIL,MOTRIN) suspension 20 mg/mL
- ☐ ibuprofen (MOTRIN) chewable tablet 100 mg
- ☐ ibuprofen (MOTRIN) tablet 200 mg

Cough Suppressants

- ☐ benzonatate (TESSALON) capsule 100 mg
- ☐ benzonatate (TESSALON) capsule 200 mg
- ☐ dextromethorphan polistirex ER (DELSYM) suspension 6 mg/mL
- ☐ dextromethorphan polistirex ER (DELSYM) suspension 6 mg/mL
- ☐ dextromethorphan polistirex ER (DELSYM) suspension 6 mg/mL

Bronchospasm Management

- ☐ albuterol (PROAIR HFA ; PROVENTIL HFA ; VENTOLIN HFA) inhaler 108 (90 BASE) MCG/ACT
- ☐ albuterol nebulizer solution 2.5 mg/3 mL (0.083%)
- ☐ VENTOLIN HFA inhaler 108 (90 BASE) MCG/ACT - Medicaid Preferred

Pediatric Acute Rhinosinusitis Supportive Care

Antipyretics & Pain Management

- ☐ acetaminophen (TYLENOL) chewable tablet 160 mg
- ☐ acetaminophen (TYLENOL) liquid 160 mg/5mL
- ☐ acetaminophen (TYLENOL) tablet 325 mg
- ☐ ibuprofen (ADVIL,MOTRIN) suspension 20 mg/mL
- ☐ ibuprofen (MOTRIN) chewable tablet 100 mg
- ☐ ibuprofen (MOTRIN) tablet 200 mg

Decongestants

- ☐ oxymetazoline (AFRIN) nasal spray 0.05%
- ☐ pseudoephedrine (SUDAFED) tablet 30 mg

Mucolytics

- ☐ guaiFENesin (ROBITUSSIN) liquid 100 mg/5mL
- ☐ guaiFENesin (ROBITUSSIN) liquid 100 mg/5mL
- ☐ guaiFENesin tablet 200 mg
- ☐ guaiFENesin (HUMIBID E) tablet 400 mg

Antihistamines

- ☐ cetirizine (ZyrTEC) chewable tablet 5 mg
- ☐ cetirizine (ZyrTEC) syrup 1 mg/mL
- ☐ cetirizine (ZyrTEC) syrup 1 mg/mL
- ☐ cetirizine (ZyrTEC) tablet 10 mg
- ☐ loratadine (CLARITIN) chewable tablet 5 mg
- ☐ loratadine (CLARITIN) syrup 5 mg/5 mL
- ☐ loratadine (CLARITIN) tablet 10 mg

Cough Suppressants

- ☐ benzonatate (TESSALON) capsule 100 mg
- ☐ benzonatate (TESSALON) capsule 200 mg
- ☐ dextromethorphan polistirex ER (DELSYM) suspension 6 mg/mL
- ☐ dextromethorphan polistirex ER (DELSYM) suspension 6 mg/mL
- ☐ dextromethorphan polistirex ER (DELSYM) suspension 6 mg/mL

benzonatate (TESSALON) 100 mg capsule

✔ Accept | ✖ Canc

Sig Method: **Specify Dose, Route, Frequency** | Taper/Ramp | Combination Dosage | Use Free Text

Dose: 100 mg | **100 mg** | 200 mg

Calculated dose: 1 capsule

Route: **oral**

Frequency: 3 times daily PRN | **TID PRN**

PRN Reasons: ✓ cough

PRN Comment: Max 600mg (6 capsules) in 24 hours.

Duration: 10 | Doses | **Days** | 30 days | 2 months | 3 months | 6 months | 1 year

Starting: 4/2/2025 Ending: 4/12/2025 First fill:

Dispense: Days/Fill: Full (10 Days) | 30 Days | 90 Days

Quantity: 30 capsule Refill: 0 | **0** | 1 | 2 | 3 | 11

Supplementary Figure 2 – Non-Pharmacologic Supportive Care SmartPhrases

Educational information to provide in "Patient Instructions" of After Visit Summary for Encounter

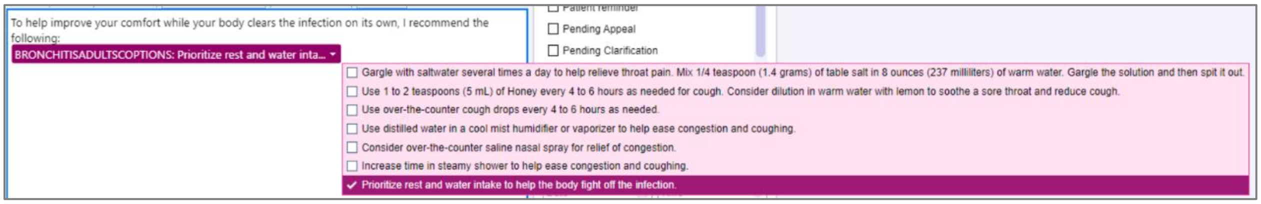

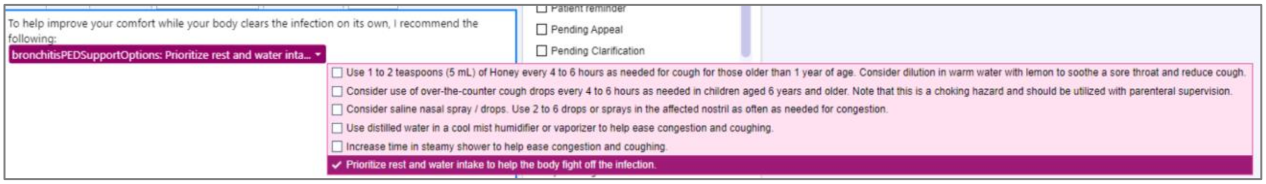

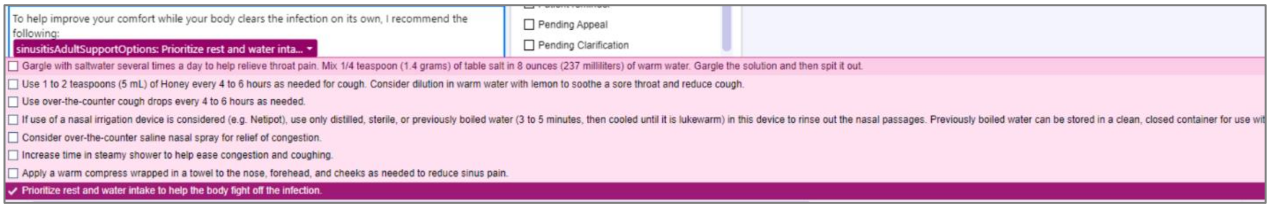

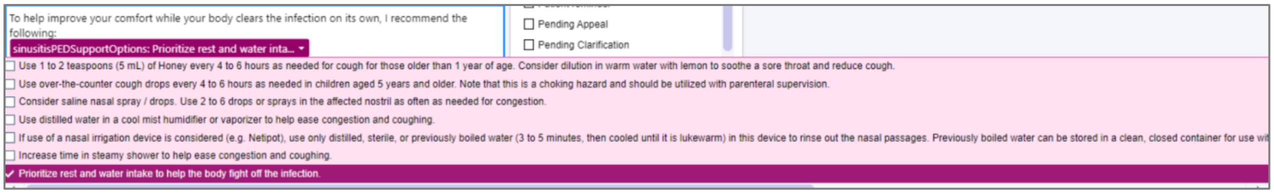

Supplementary Figure 3 – Patient Facing Flyer

What are antibiotic-resistant bacteria?

Antibiotic resistance occurs when bacteria no longer respond to the drugs designed to kill them. Anytime antibiotics are used, they can cause antibiotic resistance.

Bacteria, not the body, become resistant to the antibiotics designed to kill them.

When bacteria become resistant, antibiotics cannot fight them, and the bacteria multiply.

Some resistant bacteria can be harder to treat and can spread to other people.

Each year in the United States, **at least 2.8 million people** become infected with antibiotic-resistant bacteria. At least 35,000 people die as a result.

Can I feel better without antibiotics?

Respiratory viruses typically go away in a week or two **without treatment**. To stay healthy and keep others healthy, you can:

- Clean hands
- Cover coughs
- Stay home when sick
- Get recommended vaccines

Improve antibiotic use to combat antibiotic resistance

Centers for Disease Control (CDC) is working to reduce unnecessary antibiotic use.

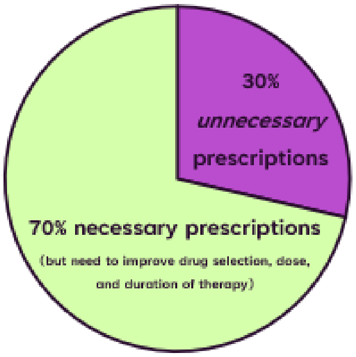

The 2020-2025 White House National Action Plan to Combat Antibiotic-Resistant Bacteria (CARB) aims to lower the annual rate of outpatient antibiotic dispensing per 1,000 U.S. population among specified subpopulations.

To learn more about antibiotic prescribing and use, visit www.cdc.gov/antibiotic-use

CDC. Outpatient Antibiotic Prescribing in the United States. Antibiotic Prescribing and Use. Published June 28, 2024.
CDC. About Antimicrobial Resistance Investments & Action. Antimicrobial Resistance. Published May 7, 2024.
